# The Burden of Periprosthetic Fractures Following Total Hip and Knee Arthroplasty: Risk Factors, Trends, and Outcomes

**DOI:** 10.7759/cureus.106534

**Published:** 2026-04-06

**Authors:** Matthew A Peterman, Jane C Brennan, Andrea H Johnson, Justin Turcotte, Paul J King, James MacDonald

**Affiliations:** 1 Orthopedic Research, Anne Arundel Medical Center, Annapolis, USA; 2 Orthopedics, Anne Arundel Medical Center, Annapolis, USA; 3 Orthopedic and Surgical Research, Anne Arundel Medical Center, Annapolis, USA; 4 Orthopedic Surgery, Anne Arundel Medical Center, Annapolis, USA

**Keywords:** clinical outcomes, national claims database, periprosthetic fracture, risk factors, total hip arthroplasty, total knee arthroplasty

## Abstract

Background

Periprosthetic fractures (PPFs) are a significant complication following total hip arthroplasty (THA) and knee arthroplasty (TKA), resulting in substantial patient morbidity. This study aims to quantify the burden of PPF, describe surgical management trends, and identify key patient risk factors in the United States population.

Methods

A retrospective review of patients undergoing primary unilateral THA or TKA from 2016 to 2022 was performed using the PearlDiver national database. The primary outcome was the burden of PPF in both THA and TKA. Multivariate analyses were performed to identify risk factors for PPF, and univariate analyses were performed to compare outcomes across surgical treatments: open reduction and internal fixation (ORIF), revision, and revision + ORIF combined.

Results

From 2016 to 2022, the burden of THA PPFs increased from 884 (1.35%) to 1,622 (1.50%), an 11.1% relative increase. The burden of TKA PPFs increased from 669 (0.55%) to 1,147 (0.61%), a 10.9% relative increase. In both populations, increased age, female sex, and higher comorbidity burden were associated with increased risk of PPF. The most common fixation method of PPF after THA was revision, with 2,712 (77.6%) cases, followed by revision + ORIF with 489 (14.0%) cases and ORIF with 295 (8.4%) cases. The most common fixation method of PPF after TKA was revision, with 856 (51.5%) cases, followed by ORIF with 767 (46.2%) cases and revision + ORIF with 38 (2.3%) cases. High 90-day complication rates were observed across treatments.

Conclusion

The burden of PPFs after THA and TKA increased by approximately 11% from 2016 to 2022, although this did not achieve statistical significance. Targeted strategies for preventing PPFs in high-risk patients and further investigation into optimal treatment methods are warranted, given the high morbidity associated with these injuries.

## Introduction

Periprosthetic fracture (PPF) is a severe and increasingly common complication after total hip arthroplasty (THA) and total knee arthroplasty (TKA), reflecting both the growing volume of joint arthroplasties performed and an aging, comorbid patient population [1,2]. Recent registry data indicate that PPF is one of the top three indications for revision after THA [1,3]. International registry and multicenter data report the worldwide annual risk of PPF to be 0.04-0.2% per year following THA and 0.03-0.25% per year following TKA [1,3-7]. In the United States, large cohort and claims database studies have reported similar annual rates of PPF after THA, at 0.1-0.2% per year, and 0.05-0.15% per year following TKA [1,2,8]. National data from the US and Europe consistently demonstrate increasing annual rates, with projections indicating that the overall burden of PPF following primary THA or TKA will increase as arthroplasty volumes escalate and populations age [2,8]. The economic burden following PPF is also substantial, with patients experiencing longer hospitalizations, increased readmission rates, and significantly higher healthcare costs compared with primary arthroplasty or native hip fractures [6,9,10]. Recovery is often prolonged and associated with substantial loss of independence and an increased need for long-term care [6,10,11]. With increasing arthroplasty utilization and expanding indications, these burdens on healthcare systems are expected to increase over the next decade [2,8,12].

Previously described risk factors for PPF include advanced age, female sex, osteoporosis, previous fragility fractures, and inflammatory arthropathies [2,6,7,13,14]. Higher overall comorbidity burden and frailty have been consistently shown in large cohort studies to confer significantly increased susceptibility to PPF [3,15-19]. Management of PPF is guided by fracture location, implant stability, and underlying bone quality, and is most commonly guided by the Vancouver classification system (VCS) for proximal femur PPFs and the Lewis and Rorabeck classification alongside the Su classification for distal femur PPFs [1,6,7]. The main surgical fixation strategies for these fractures are open reduction and internal fixation (ORIF), which is preferred for fractures around well-fixed implants, and revision with or without internal fixation, preferred when implants are loose or unstable or when bone stock is compromised [1,20]. In the US, recent national database studies have consistently reported that THA-associated PPFs are most commonly managed with revision, and TKA-associated PPFs are most frequently managed with ORIF, although recent trends indicate a rise in the use of ORIF for select THA cases [6,8]. A consistent finding throughout the literature is the high burden of morbidity and mortality among patients who experience PPF [9]. Large population-based studies show consistently poor outcomes following PPF, with one-year mortality rates reported to be between 10% and 24% and five-year mortality rates often exceeding 50% or more, comparable to native hip fracture outcomes [4,6,9]. Major complications are common, including functional decline, reoperation rates of 10-32%, and infection rates ranging from 4% to 14% [6,8,10].

Despite advances in arthroplasty and increasing experience with PPFs, management remains challenging. Patients continue to experience high complication, readmission, and mortality rates [4,6,9], and no clear consensus exists regarding the optimal surgical approach for certain fracture subtypes and complex clinical presentations [8,12,20]. The objective of the present study is to leverage a large-scale national database to identify the burden of PPF, describe trends in surgical management of PPF after THA and TKA, and identify key patient risk factors for PPF in a population that underwent THA or TKA during 2016-2022.

## Materials and methods

This study was deemed exempt by the institutional review board as a retrospective review of a de-identified database.

Data source

The PearlDiver (PearlDiver Inc., Colorado Springs, Colorado; www.pearldiverinc.com) Mariner 170 dataset was retrospectively analyzed. The database contains claims records from over 170 million patients across all payers, including commercial, Medicare, Medicaid, and self-pay. Data are searchable by International Classification of Diseases, Ninth Revision (ICD-9), International Classification of Diseases, Tenth Revision (ICD-10), and Current Procedural Terminology (CPT) codes [21].

Study population

All patients included in this study had a primary THA or TKA between 2016 and 2022. Patients with unknown laterality and those with a hip fracture on the same day as THA were excluded. Patients were grouped by whether they had a PPF after TJA.

Independent variables

The following demographics and comorbidities (based on ICD-9/10 codes) were extracted and used: age, Charlson comorbidity index (CCI) score, gender, osteoporosis, rheumatoid arthritis, cognitive disorders, dyslipidemia, stroke, malnutrition, tobacco use, alcohol disorders, cardiac disorders, diabetes, obesity, adrenal corticosteroid use, osteoarthritis, and osteonecrosis.

Outcome measures

The primary outcome of interest was the burden of PPF in both THA and TKA. The PPF burden is defined as the number of PPFs occurring in a given year, expressed as a percentage of primary THAs or TKAs performed in that year. Secondary outcomes included the identification of risk factors associated with PPF after THA and TKA. Of note, osteonecrosis was not assessed as a risk factor after TKA due to insufficient numbers of patients with osteonecrosis in the dataset. Additionally, 90-day postoperative outcomes and complications were analyzed among patients with PPF, stratified by fixation method: revision arthroplasty, open reduction and internal fixation (ORIF), or combined revision and ORIF. Evaluated complications included hospital readmission, periprosthetic joint infection (PJI), pulmonary embolism (PE), deep vein thrombosis (DVT), pneumonia, respiratory failure, urinary tract infection (UTI), myocardial infarction (MI), stroke, and atrial fibrillation.

Statistical analysis

Multivariate logistic regression was used to assess risk factors for PPF in both THA and TKA. Univariate analyses (chi-square and independent samples t-tests) were performed to compare outcomes by fixation methods. Where the assumptions of the chi-square test were not met, the Fisher-Freeman-Halton exact test was used. All statistical analyses were performed within the PearlDiver platform using R (R Studio PBC, Boston, Massachusetts). Statistical significance was assessed at p<0.05.

## Results

**Figure 1:**
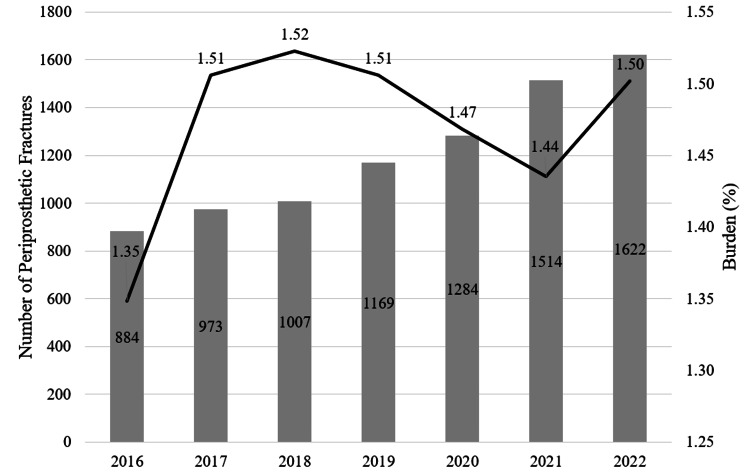
Burden of Periprosthetic Fracture in Total Hip Arthroplasty The burden of periprosthetic fracture in total hip arthroplasty increased from 1.35% to 1.50% over the study period, an 11.1% relative increase (p = 0.213). The bar graph portion is referenced on the left axis and represents the overall number of periprosthetic fractures in each given year. The line graph portion is referenced on the right axis and represents the burden of periprosthetic fractures per year (burden = total number of periprosthetic fractures/total number of primary total hip arthroplasties performed).

From 2016 to 2022, the number of PPF increased from 884 (1.35%) to 1,622 (1.50%) following THA, an 11.1% relative increase (p = 0.213) (Figure 1).

**Figure 2:**
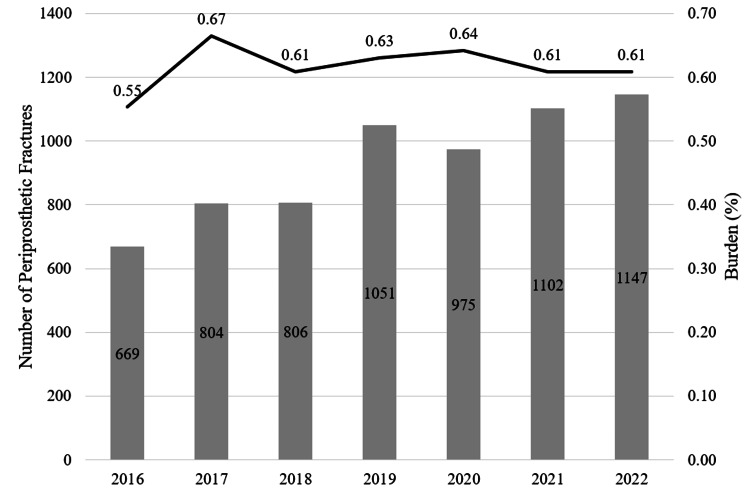
Burden of Periprosthetic Fracture in Total Knee Arthroplasty The burden of periprosthetic fracture in total knee arthroplasty increased from 0.55% to 0.61% over the study period, a 10.9% relative increase (p = 0.054). The bar graph portion is referenced on the left axis and represents the overall number of periprosthetic fractures in each given year. The line graph portion is referenced on the right axis and represents the burden of periprosthetic fractures per year (burden = total number of periprosthetic fractures/total number of primary total knee arthroplasties performed).

The number of PPF in TKA increased from 669 (0.55%) to 1,147 (0.61%) following TKA, a 10.9% relative increase (p = 0.054) (Figure 2).

The average time to PPF was 674.7 ± 647.9 days following THA and 556.4 ± 610.8 days following TKA. Older patients (OR: 1.03, 95% CI: 1.02 to 1.03; p<0.001), women (OR: 1.61, 95% CI: 1.52 to 1.69; p<0.001), and those with an increased comorbidity burden (OR: 1.07, 95% CI: 1.06 to 1.08; p<0.001) had increased odds of PPF following THA. Of the 14 individual comorbidities assessed, seven were associated with increased odds of PPF. Notably, alcohol disorders (OR: 2.08; 95% CI: 1.91 to 2.26; p<0.001), osteonecrosis (OR: 1.58, 95% CI: 1.45 to 1.72; p<0.001), cognitive disorders (OR: 1.32, 95% CI: 1.17 to 1.48; p<0.001), and malnutrition (OR: 1.21, 95% CI: 1.03 to 1.41; p=0.015) were associated with the highest odds of PPF (Table 1).

**Table 1:** Risk Factors of Periprosthetic Fracture in Total Hip Arthroplasty P-value < 0.05 in bold. CCI, Charlson Comorbidity Index; CI, confidence interval.

Risk Factor	Odds Ratio	95% CI	P-Value
Age, years	1.03	1.02 to 1.03	<0.001
CCI score	1.07	1.06 to 1.08	<0.001
Female	1.61	1.52 to 1.69	<0.001
Osteoporosis	1.36	1.27 to 1.45	<0.001
Rheumatoid arthritis	1.14	1.04 to 1.24	0.004
Cognitive disorders	1.32	1.17 to 1.48	<0.001
Dyslipidemia	0.88	0.83 to 0.92	<0.001
Stroke	0.99	0.91 to 1.07	0.767
Malnutrition	1.21	1.03 to 1.41	0.015
Tobacco use	1.18	1.12 to 1.25	<0.001
Alcohol disorders	2.08	1.91 to 2.26	<0.001
Cardiac disorders	1.02	0.96 to 1.07	0.554
Diabetes	0.93	0.88 to 0.99	0.023
Obesity	1.02	0.97 to 1.08	0.394
Adrenal corticosteroid use	0.96	0.91 to 1.02	0.200
Osteoarthritis	1.04	0.95 to 1.14	0.407
Osteonecrosis	1.58	1.45 to 1.72	<0.001

Similarly, in TKA, older patients (OR: 1.01, 95% CI: 1.01 to 1.02; p<0.001), women (OR: 2.00, 95% CI: 1.88 to 2.17; p<0.001), and those with an increased comorbidity burden (OR: 1.11, 95% CI: 1.10 to 1.13; p<0.001) were at increased odds of PPF. Of the nine significant comorbidities associated with increased odds of PPF, alcohol disorders (OR: 1.88, 95% CI: 1.66 to 2.11; p<0.001), malnutrition (OR: 1.59, 95% CI: 1.32 to 1.92; p<0.001), osteoporosis (OR: 1.48, 95% CI: 1.37 to 1.60; p<0.001), and cognitive disorders (OR: 1.34, 95% CI: 1.16 to 1.54; p<0.001) were associated with the highest odds (Table 2).

**Table 2:** Risk Factors of Periprosthetic Fracture in Total Knee Arthroplasty P-value < 0.05 in bold. CCI, Charlson Comorbidity Index; CI, confidence interval.

Risk Factor	Odds Ratio	95% CI	P-Value
Age	1.01	1.01 to 1.02	<0.001
CCI score	1.11	1.10 to 1.13	<0.001
Female	2.00	1.88 to 2.17	<0.001
Osteoporosis	1.48	1.37 to 1.60	<0.001
Rheumatoid arthritis	1.21	1.10 to 1.34	<0.001
Cognitive disorders	1.34	1.16 to 1.54	<0.001
Dyslipidemia	0.81	0.76 to 0.87	<0.001
Stroke	0.97	0.89 to 1.07	0.591
Malnutrition	1.59	1.32 to 1.92	<0.001
Tobacco use	1.19	1.11 to 1.27	<0.001
Alcohol disorders	1.88	1.66 to 2.11	<0.001
Cardiac disorders	1.14	1.07 to 1.21	<0.001
Diabetes	1.09	1.02 to 1.17	0.013
Obesity	1.03	0.97 to 1.10	0.366
Adrenal corticosteroid use	0.87	0.81 to 0.94	<0.001
Osteoarthritis	1.05	0.93 to 1.20	0.424

The most common fixation method of PPF after THA was revision, with 2,712 (77.6%) cases, followed by revision with ORIF with 489 (14.0%) cases, and ORIF with 295 (8.4%) cases. Rates of 90-day postoperative PJI varied significantly by fixation type (p = 0.002), with the highest rate observed in patients who underwent revision, with 254 (9.4%) cases (Table 3).

**Table 3:** Ninety-Day Outcomes by Periprosthetic Fracture Fixation Method in Total Hip Arthroplasty P-Value < 0.05 in bold. All data presented as n (%); *X^2^* indicates chi-square test of independence. * Fisher-Freeman-Halton Exact test, where the assumptions of the chi-square test were not met. ORIF, open reduction internal fixation.

Outcome	Revision (n = 2,712)	ORIF (n = 295)	Revision+ ORIF (n = 489)	Test Statistic	P-value
Readmission	536 (19.8)	52 (17.6)	82 (16.8)	*X^2 ^*= 2.89	0.236
Prosthetic joint infection	254 (9.4)	10 (3.3)	38 (7.8)	*X^2 ^*= 12.58	0.002
Pulmonary embolism	5 (0.2)	0 (0)	1 (0.2)		1*
Deep vein thrombosis	80 (2.9)	9 (3.1)	15 (3.1)	*X^2 ^*= 0.03	0.987
Pneumonia	102 (3.8)	11 (3.7)	19 (3.9)	*X^2 ^*= 0.02	0.990
Respiratory failure	152 (5.6)	13 (4.4)	34 (7.0)	*X^2 ^*= 2.39	0.302
Urinary tract infection	295 (10.9)	28 (9.5)	57 (11.7)	*X^2 ^*= 0.89	0.641
Myocardial infarction	44 (1.6)	3 (1.0)	7 (1.4)		0.830*
Stroke	42 (1.5)	4 (1.4)	6 (1.2)		0.940*
Atrial fibrillation	289 (10.7)	38 (12.9)	49 (10.0)	*X^2 ^*= 1.69	0.429

The most common fixation method of PPF after TKA was revision, with 856 (51.5%) cases, followed by ORIF with 767 (46.2%) cases, and revision with ORIF with 38 (2.3%) cases. Rates of 90-day postoperative readmission and PJI varied significantly by fixation type (both p<0.001), with the highest rates observed in patients who underwent revision (readmission: 165 (19.3%), PJI: 125 (14.7%)). Additionally, there were differences in rates of DVT (p=0.012), with the highest rate observed in patients who underwent revision with ORIF, with 4 (10.5%) cases (Table 4).

**Table 4:** Ninety-Day Outcomes by Periprosthetic Fracture Fixation Method in Total Knee Arthroplasty P-value < 0.05 in bold. All data presented as n (%); *X^2^* indicates chi-square test of independence. *Fisher-Freeman-Halton Exact test, where the assumptions of the chi-square test were not met. ORIF, open reduction internal fixation.

Outcome	Revision (n = 856)	ORIF (n = 767)	Revision+ ORIF (n = 38)	Test Statistic	P-value
Readmission	165 (19.3)	80 (10.4)	6 (15.8)	*X^2 ^*= 24.86	<0.001
Prosthetic joint infection	126 (14.7)	29 (3.8)	3 (7.9)	*X^2 ^*= 56.35	<0.001
Pulmonary embolism	5 (0.6)	3 (0.4)	0 (0)		0.774*
Deep vein thrombosis	31 (3.6)	17 (2.2)	4 (10.5)		0.012*
Pneumonia	37 (4.3)	21 (2.7)	2 (5.3)		0.171*
Respiratory failure	64 (7.5)	47 (6.1)	1 (2.6)		0.350*
Urinary tract infection	91 (10.6)	78 (10.2)	5 (13.2)	*X^2 ^*= 0.39	0.823
Myocardial infarction	16 (1.9)	16 (2.1)	1 (2.6)		0.948*
Stroke	23 (2.7)	16 (2.1)	0 (0)		0.481*
Atrial fibrillation	106 (12.4)	99 (12.9)	8 (21.1)	*X^2 ^*= 2.46	0.293

## Discussion

PPFs continue to represent a serious complication of hip and knee arthroplasty, associated with significant morbidity, elevated patient costs, and increased risk of revision and mortality, especially among older, frail, or medically complex patients [16]. Despite an increasing volume of primary total hip and total knee arthroplasty in the United States, the annual incidence rate of surgically treated PPFs has remained relatively stable over the last two decades when adjusted for arthroplasty volume [2,8]. From large national samples such as the Nationwide Inpatient Sample Database, the annual incidence of surgically treated PPFs has ranged between 1.84 and 2.47 per 100,000 people per year during 2006-2015 [2]. The PPF rate after primary THA is generally reported to be 0.4-3.5%, and the PPF rate after primary TKA has been reported to be 0.3-2.5% [6,10]. PPFs represent a growing share of causes for revision arthroplasty, as shown in the American Joint Replacement Registry (AJRR) database, with PPF being a leading cause of revision in elderly patients [22]. PPFs confer a similar 30-day mortality risk to native hip fracture patients; however, perioperative complication rates, both minor and major, are significantly greater in PPFs [9]. In line with other studies, our database analysis found a slight relative increase in the burden of both THA and TKA PPFs of approximately 11% over the study period, despite a significant increase in the total number of PPFs over the study period [21].

Patient demographics are consistently among the most powerful predictors of PPF risk following THA and TKA [6,13,14]. Advanced age is repeatedly cited as a primary risk factor for PPF in both THA and TKA populations across multiple large-scale registry, cohort, and database studies [13,15,16,23]. Older age exerts its effect on PPF risk through multiple mechanisms, such as age-related decline in bone quality, decreased muscle mass, frailty, and a greater incidence of falls [7,13]. Sex- and gender-related disparities have also consistently emerged in the literature, with female sex associated with heightened PPF risk after both THA and TKA in most large studies and meta-analyses. These observed differences have also been demonstrated internationally in a large Scottish national database as well as the Nordic Arthroplasty Register Association Database [18,24]. This difference can be partially attributed to the higher prevalence of osteoporosis, lower baseline bone mineral density, and greater overall frailty observed in older women [17,25]. Overall patient health status, including comorbid conditions, has been shown to impact PPF risk, with osteoporosis, osteopenia, and Charlson Comorbidity Index (CCI) having been among the most reliable predictors in both retrospective and prospective cohort analyses [6,16]. Previously described risk factors in line with this study include rheumatoid/inflammatory arthritis, chronic corticosteroid use, CKD, cognitive impairment, and previous fragility fractures [10,13,22]. Additionally, while the majority of risk factors evaluated were largely non-modifiable comorbid conditions, we observed an increased risk of PPF after both THA and TKA in patients with malnutrition, alcohol use disorders, and tobacco use, which may be targets for lifestyle modification to reduce PPF risk.

Beyond patient demographics, surgical decisions such as stem fixation method, implant design, and operative technique have been shown to directly influence postoperative fracture incidence [10,22,24,26]. Surgeons are increasingly utilizing cemented femoral stems during THA, particularly in elderly patients with poor bone quality, with the nationwide use of cemented stems rising to 5% in 2023 [22]. Coinciding with this increased utilization, large registry and cohort studies have shown a consistent reduction in PPF risk with the use of cemented femoral stems, especially in patients 65 years or older [5,16,22]. In the AJRR report, cemented fixation showed a significant reduction in revision surgeries, even after adjusting for age, sex, and CCI, in 256,108 primary THAs performed between 2012 and 2023 (HR=0.287, 95% CI, 0.192-0.43, p<0.0001) [22]. Implant design and surgical technique choices have also emerged in the literature as factors influencing PPF risk, albeit less robustly than cemented vs cementless stems. Certain stem designs are associated with disproportionate PPF risk, including those that are collarless, triple-tapered, blade type, non-grit blasted, and monoblock stems. This is potentially due to these designs having weaker metaphyseal support, resulting in stress at specific diaphyseal locations, thus increasing fracture susceptibility [19,27-29]. Modern polished taper-slip (PTS) cemented stems can also have higher PPF rates than traditional composite-beam cemented stems, as the stem can subside, leading to a wedge effect and microfracture, especially in patients with weaker bone [19,27-29]. Although more debated, anterior femoral notching has also been shown in some studies to be associated with an increased risk of periprosthetic distal femur fracture after TKA, possibly due to stress risers created by such notching [10,30]. Because our analysis utilized a national claims database, we were unable to assess these implant fixation and surgeon-specific factors in our study, highlighting the need for continued evaluation of these factors using large TJA-specific registries.

Periprosthetic femur fracture management involves two principal strategies: ORIF and revision arthroplasty. Early classification systems for proximal femur PPFs were oriented around the anatomical location of the fracture but did not consider implant stability or bone quality [1]. The Vancouver Classification System (VCS) was developed in 1995 and represented a major milestone in the systematic treatment and fixation of these PPFs, synthesizing anatomic location, implant stability, and underlying bone quality into a clinically actionable system with high inter- and intraobserver reliability [31-33]. Duncan and Masri’s VCS outlined treatment recommendations for conservative management, ORIF, revision surgery, or combinations depending on the fracture type/subgroup [1,33]. The VCS divided fractures into Type A (fractures of the trochanteric region, either the greater (AG) or lesser (AL) trochanter), Type B (fractures around or just below the stem, subdivided into B1 for well-fixed stems, B2 for loose stems with good bone stock, and B3 for loose stems with poor bone stock), and Type C (fractures well below the stem tip), considering both anatomical location and implant stability [33,34]. Most commonly, fractures were in the B category, and B1 fractures were historically well treated with ORIF, B2 necessitated revision of the femoral component due to loosening, and B3 required femoral component revision with or without proximal femoral allograft or replacement [20,32,34,35].

The vast majority of PPFs following TKA occur in the distal femur, making this region a primary concern for diagnosing and managing these fractures. Two classification systems have emerged as the most widely used and are often applied together in clinical practice. The Lewis and Rorabeck system is the most widely used and categorizes fractures based on displacement and stability of the femoral prosthesis, with type one fractures being nondisplaced about a well-fixed implant. Type two fractures are displaced, but the implant remains well-fixed, and type three fractures are displaced and associated with a loose or failing prosthesis [7]. Type one fractures are typically managed with ORIF, while implant instability or significant displacement may prompt some surgeons to favor revision arthroplasty [7,15]. The Su classification system further describes these fractures based on anatomic location, with type one fractures being proximal to the anterior flange of the femoral component, type two originating at the level of the anterior flange and extending proximally into the diaphysis, and type three extending distally into the femoral epiphysis [7]. Similar to the Vancouver classification system for proximal femur fractures, these systems help inform management between ORIF and revision, depending on fracture location, implant stability, and displacement [7].

Historically, ORIF was the predominant method for overall PPF fixation versus revision during 2006-2015, showing a declining trend from 64.4% in 2006 to 60.9% in 2015 in a nationwide dataset analysis [2]. Revision THA increased during the same period from 30.4% in 2006 to 32.5% in 2015 [2]. However, a recent national database analysis by Minutillo et al. (2016-2023) revealed divergent trends in management strategies for PPFs after THA versus TKA [8]. In TKA PPFs (n=6554), the authors observed that ORIF was the most frequently performed intervention throughout the study period, with a steadily increasing proportion of cases managed this way over time [8]. In contrast, most THA-associated PPFs were managed with revision; however, the proportion of cases managed with ORIF also increased during the study period [8]. In line with these data, our study of 8,453 patients found the most common fixation method for PPF after THA to be revision (77.6%), followed by combination revision and ORIF (14.0%) and finally ORIF (8.4%) from 2016 to 2022. However, in contrast to the Minutillo study, we found more revisions performed overall in TKA (51.5%), with ORIF (46.2%) increasing throughout the study period and rarely by combination (2.3%) [21]. Unfortunately, given the database nature of this study, we were unable to assess the specific fracture types and other clinical characteristics that influence the surgical management strategy of PPFs.

High morbidity and mortality, along with frequent and significant complications, are hallmarks of periprosthetic femur fractures following TKA and THA [4,9,11]. National database analyses estimate average one-year mortality rates to be 21-23% after PPF following THA and 21-24% after PPF following TKA [4]. Patients who experience periprosthetic lower extremity fractures also face a high complication burden, including substantial rates of fracture nonunion (1-4%), infection (5-7%), and reoperation in up to 10.9% at one year [16,20]. Most studies indicate no statistically significant difference between ORIF and revision when controlling for age, comorbidity, and fracture complexity [8,12]. Both treatments are associated with reduced mobility and quality of life compared to pre-injury status [8,11,12]. In the current literature, infection rates are similar across cohorts but may be higher with revision, typically ranging from 5% to 7% [8,12]. In our study, we observed higher 90-day prosthetic joint infection (PJI) rates in revision (13.8%) versus ORIF (4.1%) and combination revision and ORIF (10.2%) [21]. While we cannot determine causality from our database analysis, the higher 90-day PJI rate observed in the revision group may be attributable to greater surgical complexity, increased operative times, more extensive soft tissue dissection, and greater baseline patient morbidity typically associated with revision procedures [8,11,12,14].

The results of this study must be considered in the context of its limitations. As with all claims database studies, the results are dependent on the quality and accuracy of coding. Additionally, unmeasured patient- and surgeon-specific factors likely influenced both the surgical management strategies and outcomes observed. This is of particular importance in our analysis of risk factors for PPF, which was limited to coded demographics and comorbidities. Another notable limitation of this study and prior database studies is variability in methodologies employed. While other recent studies have used a cross-sectional design focused on PPF and its treatment irrespective of when the index TJA was performed, this study employs an observational cohort design [8,16]. While this enabled us to examine risk factors for PPFs, it also required that the index THA or TKA be performed within the study period of 2016 to 2022, effectively limiting our analysis to “early” PPFs, as shown by the average time to fracture of approximately two years. Therefore, direct comparisons of fracture rates and treatments across studies must be performed with caution. Finally, the assessment of outcomes in this study was limited to the short follow-up period of 90 days postoperatively and did not include important outcome measures such as functional improvement and health-related quality of life. Given the high morbidity and mortality of PPFs, evaluation of long-term outcomes remains challenging but is needed to optimize treatment selection strategies.

## Conclusions

From 2016 to 2022, the burden of THA and TKA PPFs showed a relative increase, although this did not reach statistical significance in either case. In both THA and TKA populations, older female patients with increased comorbidities were at increased risk for PPF. Surgical management strategies varied between fracture types, with THA PPFs and TKA PPFs most commonly treated with revision arthroplasty. High 90-day complication rates were observed across treatment approaches, although each approach carried varying levels of risk for different complications. These results highlight the consistent burden of PPF on the healthcare system and may inform future studies aimed at evaluating risk-reduction and treatment selection strategies.
